# Co_3_O_4_ composite nano-fibers doped with Mn^4+^ prepared by the electro-spinning method and their electrochemical properties

**DOI:** 10.1039/d0ra10336e

**Published:** 2021-07-08

**Authors:** Dasong Peng, Lianwei Duan, Xiaodong Wang, Yanchao Ren

**Affiliations:** 67th Floor, Building B, Fangyuan Building, No. 9 Ping'an Road, Luojiang District Quanzhou City Fujian Province China duanlianwei91@163.com

## Abstract

In this work, based on the electrospinning method, pure Co_3_O_4_, pure MnO_2_, and Co_3_O_4_ composite nano-fiber materials doped with different ratios of Mn^4+^ were prepared. XRD, XPS, BET and SEM tests were used to characterize the composition, structure and morphology of the materials. An electrochemical workstation was used to test the electrochemical performance of the materials. The results showed that the material properties had greatly improved on doping Mn^4+^ in Co_3_O_4_ nano-fibers. The relationship between the amount of Mn^4+^ doped in the Co_3_O_4_ composite nano-fiber material and its electrochemical performance was also tested and is discussed in this report. The results show that when *n*_Co_ : *n*_Mn_ = 20 : 2, the Co_3_O_4_ composite nano-fiber material had a specific surface area of 68 m^2^ g^−1^. Under the current density of 1 A g^−1^, the 20 : 2 sample had the maximum capacitance of 585 F g^−1^, which was obviously larger than that of pure Co_3_O_4_ nano-fibers (416 F g^−1^). After 2000 cycles of charging/discharging, the specific capacitance of the 20 : 2 sample was 85.9%, while that of the pure Co_3_O_4_ nano-fiber material was only 76.4%. The mechanism of performance improvement in the composite fibers was analyzed, which demonstrated concrete results.

## Abstract 2

In this work, based on the electrospinning method, pure Co_3_O_4_, pure MnO_2_, and Co_3_O_4_ composite nano-fiber materials doped with different ratios of Mn^4+^ were prepared.

## Graphical abstract

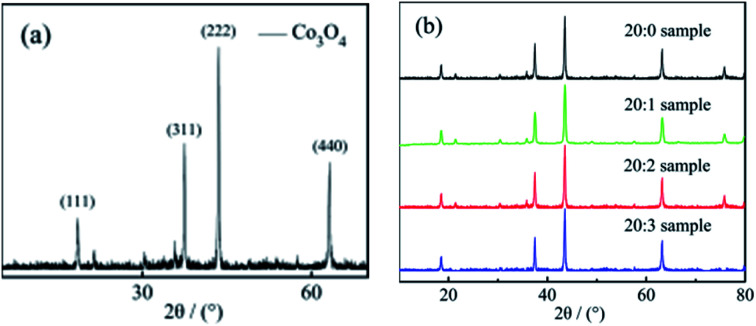

## 1. Introduction

Many investigations have been done to find clean and sustainable energy due to the global energy crisis and the aggravation of environmental pollution caused by the excessive use of fossil fuels.^1^ In many energy storage and conversion systems, supercapacitors are widely used and considered one of the most promising energy storage devices as they have the advantages of fast charging and discharging speed, high power density and long cycle stability. According to the charge storage mechanism, supercapacitors can be divided into two types: one is pseudocapacitors with redox reaction electrode materials, and the other is double-layer electric supercapacitors with carbon-based electrode materials. Electrode materials are the key components of supercapacitors. Their specific surface area, electrochemical activity and stability will directly affect the capacitance, rate performance and cycling stability, which determine their prospects in practical applications. The methods used to prepare active nano filament electrodes, such as interfacial polymerization,^2^ chemical vapor deposition,^3^ sol–gel method,^4^ and hydrothermal method have many disadvantages, such as complicated operation, low yield, high cost and difficulty in avoiding the agglomeration of nanoparticles. Electrospinning can avoid the above problems and simplify the fabrication process. It can also be used to prepare nano-fiber materials with a long one-dimensional nanostructural aspect ratio, which can increase the contact area between the electrode and the electrolyte and shorten the electron and ion transport paths, which are conducive to improve the capacitance performance of the electrode. During the charging/discharging process, pseudocapacitor electrode materials undergo volume expansion. A good mesoporous structure can prevent the structural instability caused by material expansion and impart high electric capacity, high rate performance, high energy density and excellent cycle stability in supercapacitors.

A series of studies have been performed to prepare metal oxide nano-fiber materials with various morphologies for use as capacitance electrodes, including the Co_3_O_4_ capacitance electrodes with the morphologies of nano-meter-scale flakes,^5^ three-dimensional cage,^6^ and lamellae flowers,^7^ as well as the MnO_2_ capacitance electrode that had the following morphologies: flower-like,^8^ porous,^9^ and hollow nest.^10^ However, monometallic oxide electrode materials have the drawbacks of poor structural stability and low specific capacitance capacity. To solve these problems, some new works compounded a variety of transition metal oxides together in order to enhance the structural stability of the materials, increase the ion-embedding/injection channels and efficiency, and generate the impurity band effect to improve the redox reaction efficiency of the electro-active substances.^11–13^ Huang *et al.*^14^ prepared the Co_3_O_4_/NiO/MnO_2_ ternary composite electrode material. They found that when the current density was 0.5 A g^−1^ and w_Co_3_O_4__ : w_NiO_ : w_MnO_2__ was 3 : 3 : 22, the specific capacitance capacity could reach 549 F g^−1^. Cheng *et al.*^15^ prepared a Co_3_O_4_/MnO_2_ electrode nanomaterial by a solvothermal method (core–shell grown on the surface of nickel foam). When the current density was 0.2 A g^−1^, the specific capacitance could reach 560 F g^−1^. However, this method has the drawback of a complex preparation process.

In this study, Mn^4+^-doped Co_3_O_4_ composite nanofibers were prepared by the electrospinning method, and their characteristics and electrochemical properties were tested. The results showed that the Co_3_O_4_ composite nano-fibers doped with Mn^4+^ possessed enhanced cycling charging/discharging stability as an electrode material. That is, after looping for 2000 cycles, the capacitance retention rate of pure Co_3_O_4_ was 76.4%, whereas the capacitance retention rate of the Co_3_O_4_ composite nano-fiber material doped with Mn^4+^ was 85.9%. The specific surface area of the hollow composite nano-fiber material was 68 m^2^ g^−1^, which provided a large number of activation sites for the electrochemical reaction. A hollow structure improves the utilization ratio of the material. When the current density was 1 A g^−1^, the discharging specific capacitance capacity reached 585 F g^−1^.

This report is organized as follows: in Section 2, Experimental methods, we have first explained the sample preparation process. Then, we have described the equipment used to test and characterize the structure, morphology and composition of the samples. Finally, the working electrode sample preparation and the electrochemical performance of the samples are shown. In Section 3, Results and discussion, we analyze and discuss test results in four parts. In the first part, the sample morphology and composition were analyzed by four methods: (1) XRD analysis of samples, (2) XPS analysis of samples, (3) the SEM and TEM analysis of samples, (4) the analysis of the specific surface area and the ratio of pores. In the second part, the electrochemical performances of tested samples are shown. In the third, the theoretical analyses of the electrochemical performances are shown. In the fourth, further discussions on the experimental results and analyses are presented. In Section 4, Conclusion, we draw a brief conclusion.

## 2. Experimental methods

### 2.1. Sample preparation

The experimental reagents used to prepare the samples were:

(1) HCON(CH_3_)_2_, it is abbreviated to DMF in the following text.

(2) Co(CH_3_COO)_2_·4H_2_O.

(3) Mn(CH_3_COO)_2_·4H_2_O.

(4) (C_6_H_9_NO)_*n*_, it is abbreviated to PVP in the following text.

The experimental steps used to prepare the samples were:

Step 1: 1 g Co(CH_3_COO)_2_·4H_2_O and 0.098 g Mn(CH_3_COO)_2_·4H_2_O were added to 15 mL DMF and mixed using a magnetic stirrer for 4 hours.

Step 2: 2.2 g PVP was added into the above mixture and stirred for 4 hours. Thus, the precursor of the sample was prepared.

Step 3: The precursor of the sample was into a 5 mL syringe with a needle of inner diameter 0.5 mm for wire spraying. The electrospinning apparatus used was LSP01-2A, and its static voltage was set as 15 kV; the distance between the plates was set as 20 cm.

Step 4: The spun samples were placed in a bake oven and dried for 24 hours. After that, they were placed in a sintering furnace. The temperature was increased to 600 °C at the rate of 4 °C min^−1^. The samples were maintained at 600 °C for 3 hours and cooled to room temperature naturally. After that, the *n*_Co_ : *n*_Mn_ = 20 : 2 Co_3_O_4_ composite nano-fiber material was obtained and marked as the “20 : 2 sample”.

By repeating the above steps with different mass ratios of Co(CH_3_COO)_2_·4H_2_O and Mn(CH_3_COO)_2_·4H_2_O, we prepared the *n*_Co_ : *n*_Mn_ = 20 : 1 Co_3_O_4_ composite nano-fiber material (marked as 20 : 1 sample) and *n*_Co_ : *n*_Mn_ = 20 : 3 Co_3_O_4_ composite nano-fiber material (marked as 20 : 3 sample). Meanwhile, pure Co_3_O_4_ and MnO_2_ nano-fiber materials without doping were also prepared, respectively.

### 2.2. Structure, morphology and composition of the samples

The equipment used to characterize the structure, morphology and composition were:

(1) A Bruker D8-Advance X-ray diffractometer (XRD) was used to test the phase composition of the samples. For the experiments, we selected the anode Cu target Kα radiation with an X-ray tube voltage of 40 kV and a tube current of 30 mA (Cu target, Kα radiation), and the scanning range (2*θ*) was 5°–80°.

(2) A SAM-800 photoelectron spectrometer (XPS, SAM-800) was used to analyze the surface of the samples. The electron binding energy was corrected by the C 1s peak (284.6 eV) of carbon.

(3) JSM-IT300 scanning electron microscope (accelerating voltage: 20 kV) and JEM-201 transmission electron microscope (accelerating voltage: 200 kV) were used to observe the sample morphology.

(4) A F-Sorb2400 BET instrument was used to measure the specific surface area and porosity of the samples.

### 2.3. Sample preparation and the electrochemical performance test

#### (A)Sample preparation

To test the electrochemical parameters of the samples, we used these samples to prepare working electrodes. The method to prepare the working electrodes was as follows: (1) each sample (prepared as explained in Section 2.1) was mixed with black carbon and polyfluoroethylene. The mass ratio of the three materials was *m*_sample_ : *m*_black carbon_ : *m*_polyfluoroethylene_ = 8 : 1 : 1 (w/w), and some ethanol was added into the mixture to make a slurry. (2) The slurry was coated on nickel uniformly. (3) The nickel samples were dried in a vacuum at 100 °C for 6 hours. Then, the working electrode was prepared by pressing the tablet at a pressure of 6 MPa. The 20 : 1 sample was used to prepare the electrode and marked as the 20 : 1 working electrode. The 20 : 2 sample was used to prepare the electrode and mark the 20 : 2 working electrode. The 20 : 3 sample was used to prepare the electrode and mark the 20 : 3 working electrode. The working electrode prepared using the pure Co_3_O_4_ sample nano-fiber material was marked as the pure Co_3_O_4_ working electrode and that using pure MnO_2_ was marked as the MnO_2_ working electrode.

#### (B)Auxiliary experimental materials

In the experiments, saturated calomel was used as the reference electrode and platinum as the auxiliary electrode. The electrolyte solution used in the experiments was KOH solution at a concentration of 1 mol L^−1^.

#### (C)Test equipment

The electrochemical analysis was performed using a CH1660E electrochemical workstation.

#### (D)Test parameters

The cyclic voltammetry curves and constant current charging/discharging curves of the materials were measured. The parameters set for cyclic voltammetry were as follows: the voltage range was set as 0.1–0.5 V. The scanning rates were set at 10, 20, 30, 40, and 50 mV s^−1^. The constant current charging/discharging measurement parameters were set as below: the potential window was 0–0.4 V. The current densities were 1, 2, 3, and 4 A g^−1^.

## 3. Results and Discussion

### 3.1. Sample morphology and composition

#### (A)XRD analysis of the samples

**Fig. 1:**
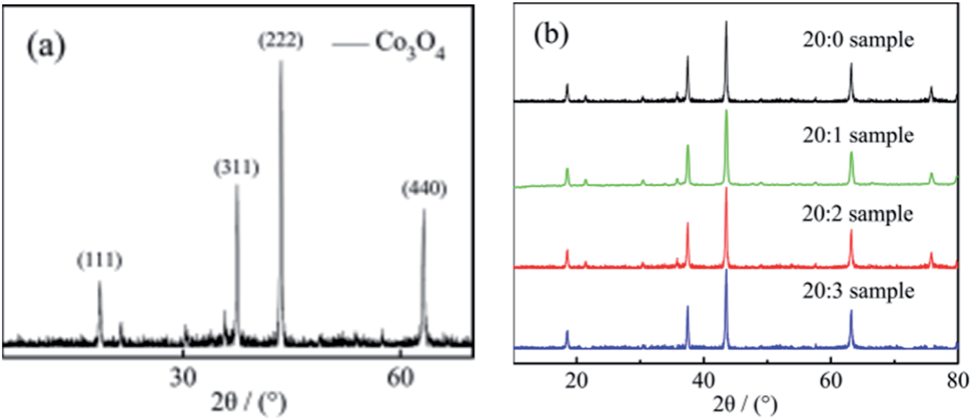
(a) The XRD test results of the pure Co_3_O_4_ working electrode sample; (b) the XRD test results of the Co_3_O_4_ composite working electrode doped with Mn^4+^.

Fig. 1 shows the XRD test results of the pure Co_3_O_4_ nano-fiber material and the three kinds of Mn^4+^-doped Co_3_O_4_ composite nano-fiber samples. From the results, it could be observed that: (1) the tour materials had the same diffraction peaks at the same scanning angles 18.8°, 31.1°, 36.7°, 37.5°, 43.5°, 63.2° and 78.1°. All these peaks were sharp. (2) A comparison of the diffraction peaks with the PDF standard card showed that the four peaks absolutely corresponded with the (111), (220), (311), (222), (111), (440) and (220) diffraction planes of Co_3_O_4_ (JCPDS no. 43-1003),^16^ respectively. (3) This indicated that the lattice structure and lattice constant of the Co_3_O_4_ crystal in the four samples were uniform.

#### (B)XPS analysis of the samples

**Fig. 2:**
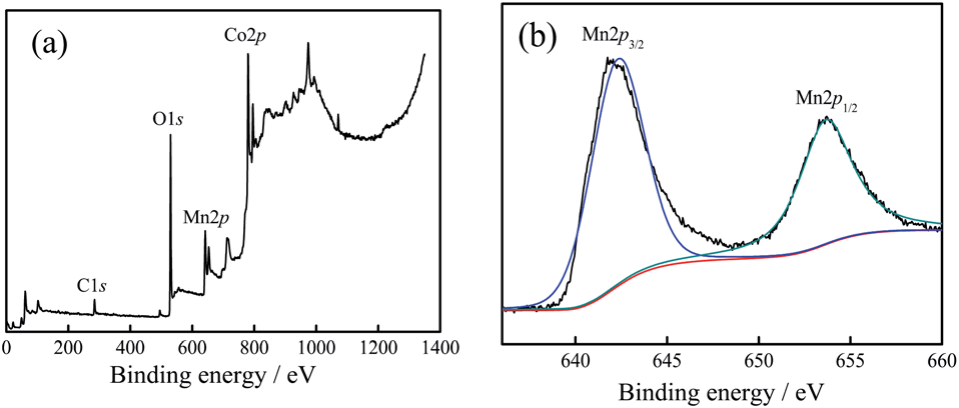
(a) XPS spectra of 20 : 2 sample, (b) Mn spectra of 20 : 2 sample.

To confirm the existence of Mn^4+^ and obtain structural information, the 20 : 2 sample was characterized in an XPS analyzer. The results are shown in Fig. 2. The binding energy of the sample was calibrated with C 1s (284.8 eV).

Fig. 2(a) shows the full spectrum analysis data, corresponding to the binding energy peaks of Co, Mn, O, C. Their proportions (*n*/*n*) were 16.73% (Co), 10.85% (Mn), 52.46% (O), 17.09% (C), respectively. The results showed that (1) the sample contained the elements Co, Mn, and O. (2) The proportion of Mn atoms was in accordance with the actually added proportion. (3) The content of Co atoms was relatively low, which was caused by the substitution of Co atoms with Mn atoms on the surface.

Fig. 2(b) shows the high-resolution XPS spectrum result of Mn. From the result, it could be inferred that: (1) the peak values for Mn 2p_3/2_ (642.2 eV) and Mn 2p_1/2_ (653.8 eV) were corresponding to the binding energy peaks of the Mn element in the Mn–O bond, showing the valence state of Mn^4+^.^17,18^ This proved that the combination of Mn^4+^ and Co_3_O_4_ nano-fibers was successfully achieved by the electrospinning method. (2) Based on the XRD test results together with the XPS characterization results, it could be judged that Mn^4+^ did not enter the crystal lattice of cobalt oxide in the composite nano-fibers, and it existed only at the interface of the nano-fiber surface and the crystal boundary. The Mn atoms replaced some of the Co atoms; one Mn^4+^ replaced two Co^2+^ atoms, and the Mn atoms combine with O atoms to form the structure of O–Mn–O. (3) In fact, the XPS characterization results revealed that the Mn atoms accounted for 10.85% (*n*/*n*), which replaced 2 × 10.85% = 21.7% Co atoms. Considering that 16.73% Co atoms still existed near the surface, the ratio *n*_Co_/*n*_O_ = (21.7% + 16.73%)/52.46% = 0.733, which was very close to the Co/O atom ratio of Co_3_O_4_ (0.75). This data supports the judgement that at the interface, Mn^4+^ substitutes some of Co^2+^, reacts with O^2−^, and forms the O–Mn–O structure.

#### (C)The SEM and TEM analysis of samples

**Fig. 3:**
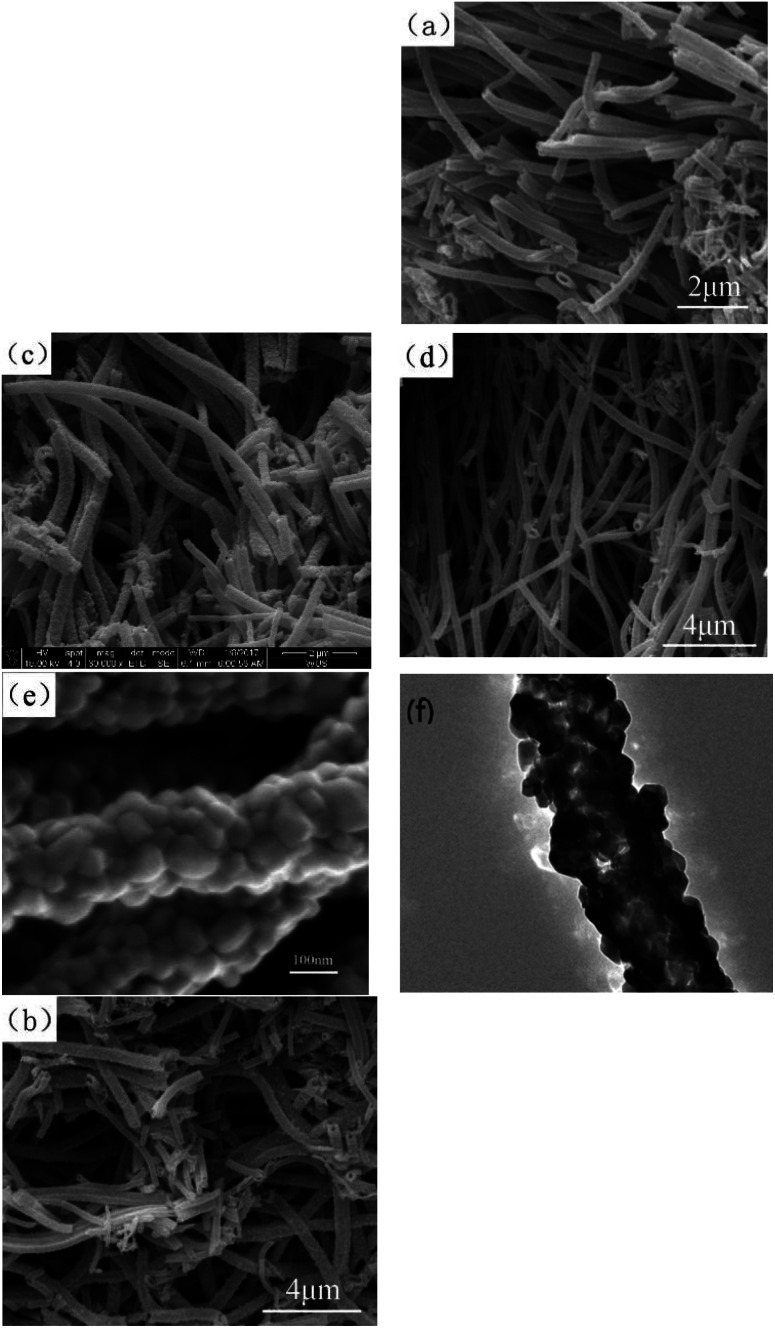
(a–c) SEM images of the pure Co_3_O_4_ nano-fibers, 20 : 1 sample, 20 : 3 sample, respectively. Fig. 3 (d–f) SEM and TEM images of the 20 : 2 sample.

Fig. 3 shows the SEM images of the 20 : 1 sample, 20 : 2 sample, 20 : 3 sample, and pure Co_3_O_4_ sample and the TEM image of the 20 : 2 sample. (1) From the SEM images, it could be seen that the lengths of the nanofibers were more than 5 μm, and their sizes were uniform; the diameter range of nanofibers was between 100 to 300 nm, and their length to diameter ratio was more than 30. (2) The surface of the composite nano-fibers was rough, as shown in Fig. 3 (b–d). (3) Fig. 3(e) clearly shows the rough surface of the 20 : 2 composite nano-fibers. The composite nano-fibers were composed of many cobalt oxide nano-particles with irregular morphology, and the size range was between 30 to 50 nm, with good crystallinity. (4) Fig. 3(f) shows the transmission electron microscopy result of the 20 : 2 sample, in which light transmission was not uniform. This indicated that there were pores left by the combustion of organic matter between the particles of the nano-fibers.

#### (D)The sample's specific surface area and the ratio of pores

**Fig. 4:**
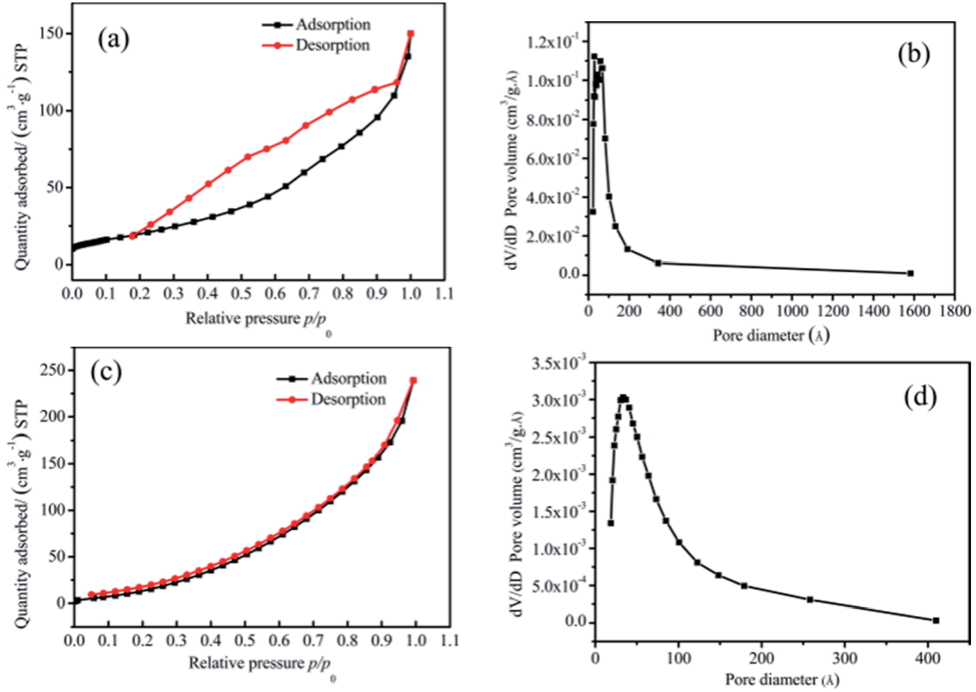
(a) and (b) The nitrogen adsorption–desorption isotherms and pore size distribution of pure Co_3_O_4_ nano-fibers, respectively. (c) and (d) The nitrogen adsorption–desorption isotherms and pore size distribution of the 20 : 2 sample.

Fig. 4 shows the isothermal adsorption and desorption curves and the pore size distribution curves of the pure Co_3_O_4_ and 20 : 2 samples. The relative pressure *p*/*p*_0_ was taken as the abscissa, where *p* is the equilibrium pressure of nitrogen, and *p*_0_ is the saturated vapor pressure of liquid nitrogen temperature, with the amount of sample adsorption and desorption as the ordinates. From Fig. 4(a) and (c), it could be seen that the interaction between the two groups of samples and the adsorbate was small, and the adsorption amount was less in the low-pressure region. With an increase in the relative pressure, the adsorption capacity also increased, which revealed that the pores were filled. When the *p*/*p*_0_ was less than 0.3, the curves of adsorption and desorption were basically coincident, which indicated that the proportion of micropores (<2 nm) was high. When the relative pressure was high, the adsorption lagged behind the desorption curve, which indicated that there were a certain number of mesopores (2–50 nm) and a small number of macropores (>50 nm).

An F-Sorb2400 instrument can automatically calculate the specific surface area. The results showed that the specific surface area of the pure Co_3_O_4_ sample was 65 m^2^ g^−1^ and that of the 20 : 2 sample was 68 m^2^ g^−1^. On comparing Fig. 4(b) with Fig. 4(d), it can be seen that the proportion of micropores and mesoporous in the 20 : 2 sample was relatively large. There were no macropores. The pore size distribution of its adsorption scale range was 2–40 nm, and its average size was about 14 nm. The surface of the pure Co_3_O_4_ sample was mainly mesoporous, with a small number of macropores. This was attributed to the presence of relevant amorphous structures formed after Mn^4+^ doping at the crystal interface of Co_3_O_4_, which would prevent organic matter from burning and forming large pores, thereby improving the structural stability of the material and bringing about a large specific surface area.

### 3.2. The electrochemical performance of the tested samples

**Fig. 5:**
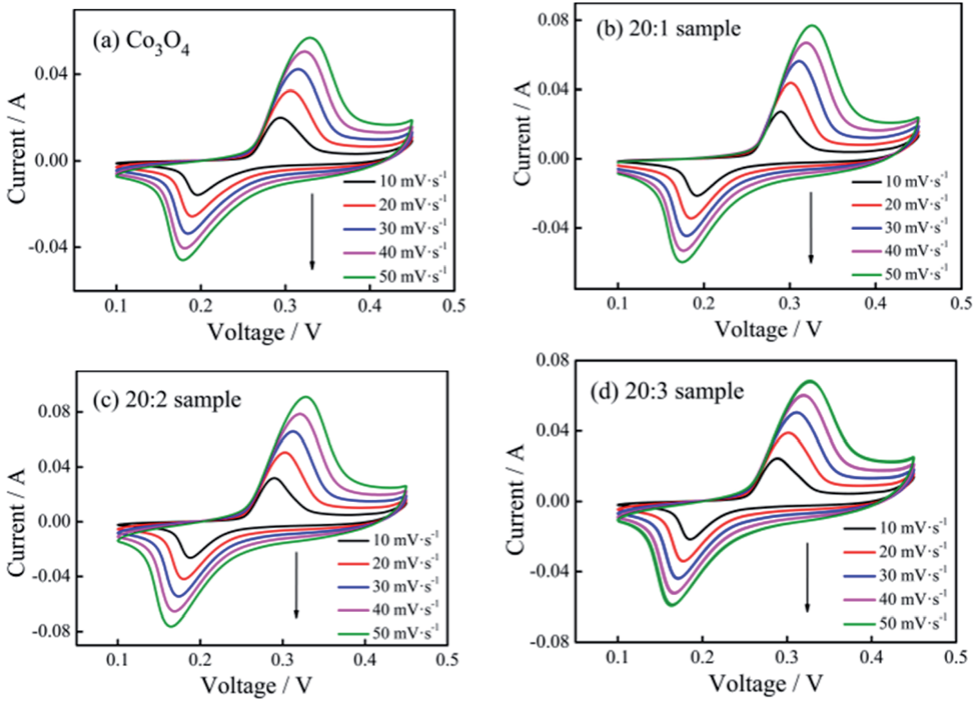
CV curves of (a) pure Co_3_O_4_ nano-fibers, (b) 20 : 1 sample, (c) 20 : 2 sample, and (d) 20 : 3 sample at different scan rates.

The cyclic voltammetry curves of the pure Co_3_O_4_ sample and composite nano-fiber samples measured at different scanning rates are shown in Fig. 5. The shapes of the curves revealed that all samples stored energy based on faradaic pseudo-capacitance, showing clear redox peaks. When the scanning rate increased, the peak current intensity increased gradually, while the peak deviation was small. The smaller offset meant better reversibility and the same redox mechanism. On comparing the closed area of their cyclic curves, it could be seen that at the same scanning rate, the 20 : 2 sample had the largest area (see Fig. 5(c)) and the maximum specific capacitance capacity.

**Fig. 6:**
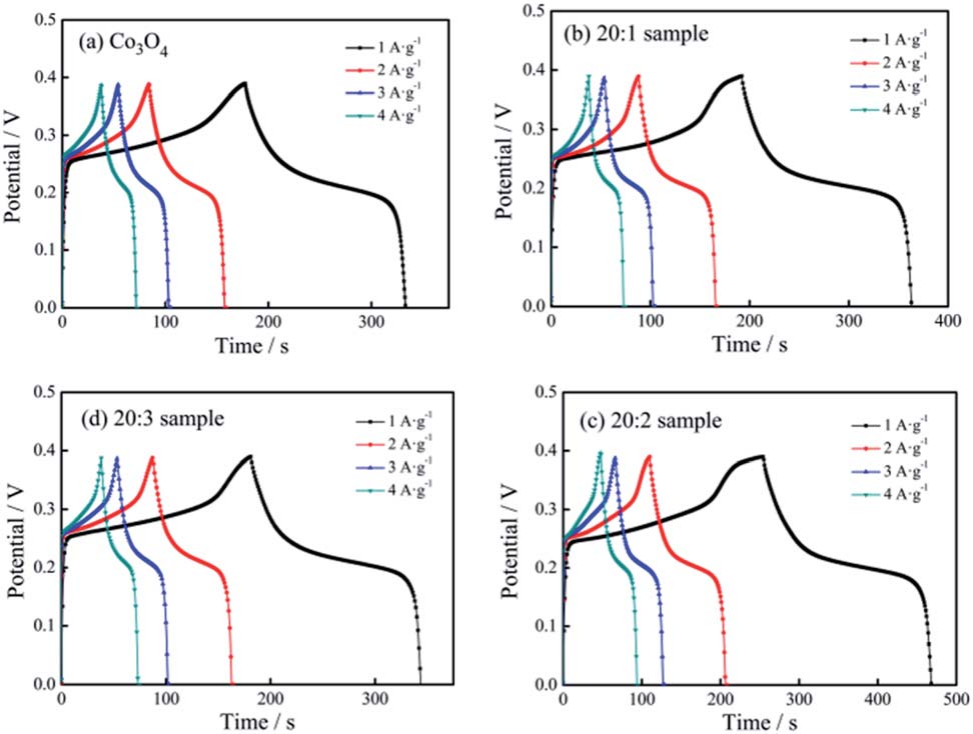
Charging/discharging curves of different electrodes: (a) pure Co_3_O_4_ nano-fibers, (b) 20 : 1 sample, (c) 20 : 2 sample, (d) 20 : 3 sample.

Fig. 6 shows the constant current charging/discharging curves of the pure Co_3_O_4_ sample, 20 : 1 sample, 20 : 2 sample and 20 : 3 sample under different current densities.

The relationships among *C*, *I*, *t*, *m*, Δ*V* can be described by the following equation:^19^

1 *C* = *I* × *t*/(*m* × Δ*V*)

where *C* is the electrochemical mass specific capacitance capacity of the electrode material (unit: F g^−1^), *I* is the discharge current (unit: A), *t* is the discharge time (unit: s), *m* is the mass of the active electrode material (unit: g), and Δ*V* is the potential window (unit: V).

Based on eqn (1), when the current densities were 1, 2, 3 and 4 A g^−1^, the specific capacitance capacity values of the pure Co_3_O_4_ sample were 416, 393, 386 and 348 F g^−1^, respectively. The values of the 20 : 1 sample were 454, 414, 384, 364 F g^−1^, respectively. The values of the 20 : 2 sample were 585, 515, 475, 469 F g^−1^, respectively, and those of the 20 : 3 sample were 429, 406, 381, 365 F g^−1^, respectively.

It could be seen that (1) with an increase in the current density, the specific capacitance of the tested samples showed a downward trend, and the extent of decline gradually decreased. (2) Because of Mn^4+^ doping, the specific capacitance capacity of the samples improved, and the specific capacitance capacity of the 20 : 2 sample showed the most obvious improvement. (3) Under the current density of 1 A g^−1^, the specific capacitance values of the 4 samples were compared, and the specific capacitance values of the 20 : 1 sample, 20 : 2 sample and 20 : 3 sample were 9.13%, 40.63% and 3.13%, respectively, and higher than that of pure Co_3_O_4_.

**Fig. 7:**
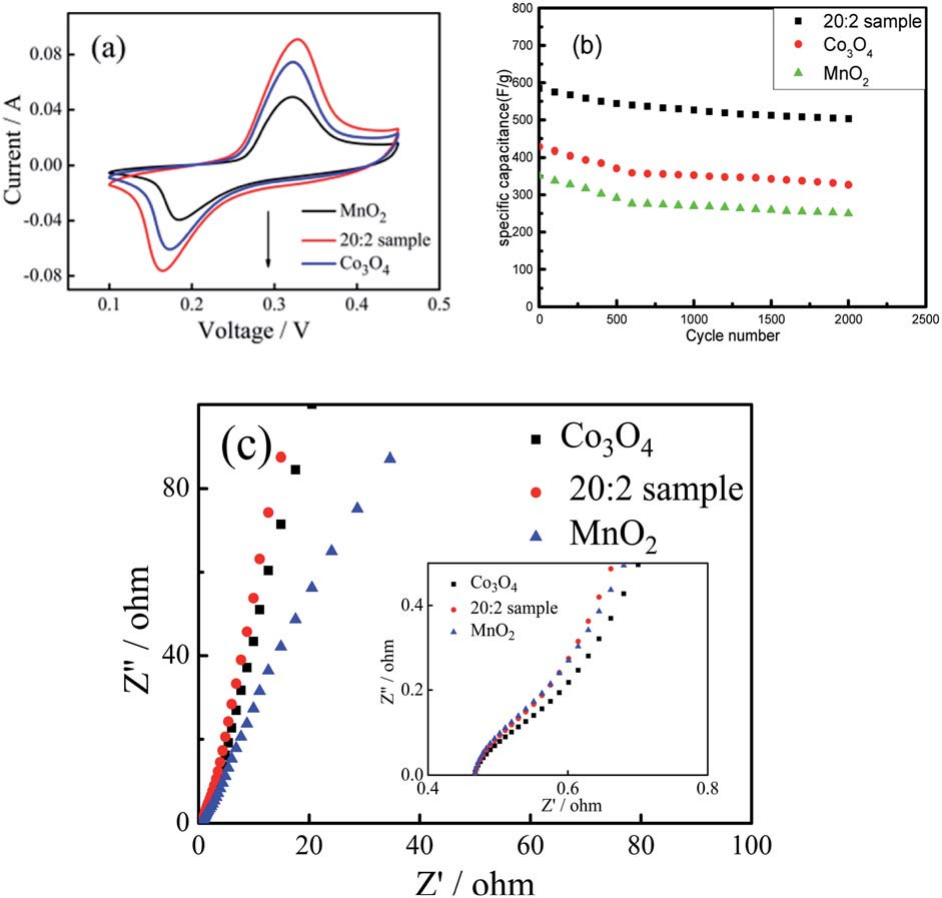
(a) CV curves for the 20 : 2 sample, pure Co_3_O_4_ nano-fibers and pure MnO_2_ nano-fibers at the scanning rate of 50 mV s^−1^. (b) Cycling stability of the 20 : 2 sample, pure Co_3_O_4_ nano-fibers and pure MnO_2_ nano-fibers at a GCD current density of 1 A g^−1^. (c) AC impedance spectra of the 20 : 2 sample, pure Co_3_O_4_ and pure MnO_2_ nano-fibers.

The cyclic voltammetric curves of the 20 : 2 sample, pure Co_3_O_4_ sample and pure MnO_2_ sample were compared and analyzed. (1) As shown in Fig. 7(a), at the scanning rate of 50 mV s^−1^, the cyclic curve area of the 20 : 2 sample was obviously larger than that of the pure Co_3_O_4_ sample and pure MnO_2_ sample, and the corresponding area of MnO_2_ was the smallest. (2) Fig. 7(b) shows the cyclic charging/discharging curves of the 20 : 2 sample, pure Co_3_O_4_ sample and pure MnO_2_ sample at 1 A g^−1^ current density. (3) After 2000 cycles, the capacitance retention rate of the 20 : 2 sample was 85.9% and those of the pure Co_3_O_4_ sample and pure MnO_2_ sample were 76.4% and 71.3%, respectively. It can be seen that the capacitance retention rate of the 20 : 2 sample was the highest.

### 3.3. Theoretical analysis of the electrochemical performance of the sample

Each Co_3_O_4_ molecule contains one Co^2+^ atom and two Co^3+^ atoms. As an electroactive substance, the electrode reaction on Co_3_O_4_ in 1 mol L^−1^ KOH alkaline medium^20^ is:

2 Co_3_O_4_ + H_2_O + OH^−^ ↔ 3CoOOH + e^−^

During charging/discharging, the corresponding redox reaction process is Co^2+^ ↔ Co^3+^ + e^−^. Here, the Co^2+^ ion in Co_3_O_4_ directly participates in the oxidation–reduction reaction and changes its valence state, which naturally affects the structural stability of Co_3_O_4_ and results in its lower specific capacitance capacity. When the Co_3_O_4_ nano-fibers are doped with Mn^4+^, it has been pointed out that at the interface of the cobalt oxide crystal, some Co^2+^ ions are replaced by Mn^4+^, and the Mn atoms combine with O atoms to form the O–Mn–O amorphous structure. This structure also participates in the charging/discharging process.^21^ The reaction can be described as:

3 MnO_2_(O–Mn–O) + K^+^ + e^−^ ↔ MnOOK

In this reaction, the valence state of Mn^4+^ remains unchanged before and after the reaction. In fact, the cyclic voltammetry curves (see Fig. 5) show that the redox peaks of all the samples were the same, indicating that the redox reaction on the samples doped with Mn^4+^ was still Co^2+^ ↔ Co^3+^ + e, and the Mn^4+^ ions did not participate in the reaction.

### 3.4. Further discussions

From the above experimental results and analysis, it can be inferred that doping Co_3_O_4_ with Mn^4+^ can improve the specific capacitance capacity and cycling stability of the composite nanofibers. The reasons can be attributed to the following aspects: (1) The O–Mn–O structure formed by Mn^4+^ doping directly participates in charging/discharging and maintains the valence state of Mn^4+^, which promotes the structural stability of the composite materials. (2) Mn^4+^ only exists at the interface, making the O–Mn–O structure on the grain surface of Co_3_O_4_ form an interface protection layer, which can prevent the structural instability caused by the change in the valence state of Co^2+^. (3) Mn^4+^ enters the gap between the Co_3_O_4_ grains and contributes to the formation of more active defect sites and ion channels in the composite nano-fiber materials, which makes more Co^2+^ participate in the charging/discharging process and improves the reaction efficiency of the active substance. (4) In addition, as shown in Fig. 7(c), the AC impedance spectra of the 20 : 2 sample, pure Co_3_O_4_ sample and pure MnO_2_ sample were similar. Moreover, the electronic conduction resistance was also similar. However, in the low-frequency region, the slope of the oblique line for the 20 : 2 sample was larger than that of the pure Co_3_O_4_ and MnO_2_ samples. This indicates that the ionic diffusion resistance of the composite sample was less than that of the pure oxide sample. This is the direct positive effect of Mn^4+^ doping. If the amount of Mn^4+^ doped in the Co_3_O_4_ composite nano-fibers is too large, the surplus Mn^4+^ cannot enter into the O–Mn–O structure, which will weaken the charging/discharging reaction efficiency of the composite. However, if the amount of Mn^4+^ doped in the Co_3_O_4_ nano-fibers is too small, it will not be conducive to fully realizing the effect of Mn^4+^ doping, that is, to stabilize the structure of Co_3_O_4_ and promote the improvement of reaction efficiency.

## 4. Conclusions

In this work, by the electrospinning method and calcination at 600 °C, three kinds of Mn^4+^-doped Co_3_O_4_ composite nano-fiber samples were prepared. The *n*_Co_ : *n*_Mn_ of the three samples were 20 : 1, 20 : 2 and 20 : 3, respectively. The nano-fiber materials had a hollow structure. The specific surface area of the 20 : 2 sample reached 68 m^2^ g^−1^. From the pore size distribution curves, it was observed that the proportion of micropores and macropores on the surface of the sample was small, while the proportion of mesopores was large, and the average pore size for adsorption was about 14 nm.

The electrochemical performance test results showed that the samples had clear redox peaks, among which the 20 : 2 sample had the maximum discharging specific capacitance capacity, and the capacity was 585 F g^−1^ at the current density of 1 A g^−1^. After 2000 cycles, the specific capacitance retention rate of the sample was 85.9%, which was obviously better than those of the pure Co_3_O_4_ sample and pure MnO_2_ sample.

The reasons for the improvement in the electrochemical properties of the composite nano-fiber materials are as follows: (1) the large specific surface area of the hollow composite nano-fibers provides sufficient activation sites for the electrochemical reaction. (2) By substituting Co^2+^ on the surface, Mn^4+^ in the composite can form the O–Mn–O bond, which can participate in the charging/discharging process and exists at the Co_3_O_4_ interface in the amorphous form to maintain the stability of the crystal structure. (3) The composite nano-fibers with doped Mn^4+^ produce more ion channels, which are conducive to reducing the ion diffusion resistance.

Subsequently, for our next report, Ni^2+^-doped Co_3_O_4_ composite nano-fibers will be prepared by electrospinning to test their electrochemical properties, and a ternary composite nano-fiber material will be prepared to explore the mechanism of the stable structure.

## Conflicts of interest

There are no conflicts to declare.

## Supplementary Material
