# Correction: Co_3_O_4_ composite nano-fibers doped with Mn^4+^ prepared by the electro-spinning method and their electrochemical properties

**DOI:** 10.1039/d1ra90132j

**Published:** 2021-08-03

**Authors:** Dasong Peng, Lianwei Duan, Xiaodong Wang, Yanchao Ren

**Affiliations:** Quanzhou Yunjian Measurement Control and Perception Technology Innovation Research Institute, 67th Floor, Building B, Fangyuan Building, No. 9 Ping’an Road, Luojiang District Quanzhou City Fujian Province China duanlianwei91@163.com

## Abstract

Correction for ‘Co_3_O_4_ composite nano-fibers doped with Mn^4+^ prepared by the electro-spinning method and their electrochemical properties’ by Dasong Peng *et al.*, *RSC Adv.*, 2021, **11**, 24125–24131, DOI: 10.1039/D0RA10336E.

The authors regret that an incorrect version of Fig. 3 was included in the original article, as the panels were shown in an incorrect order. The correct version of Fig. 3 is presented below.

**Fig. 3:**
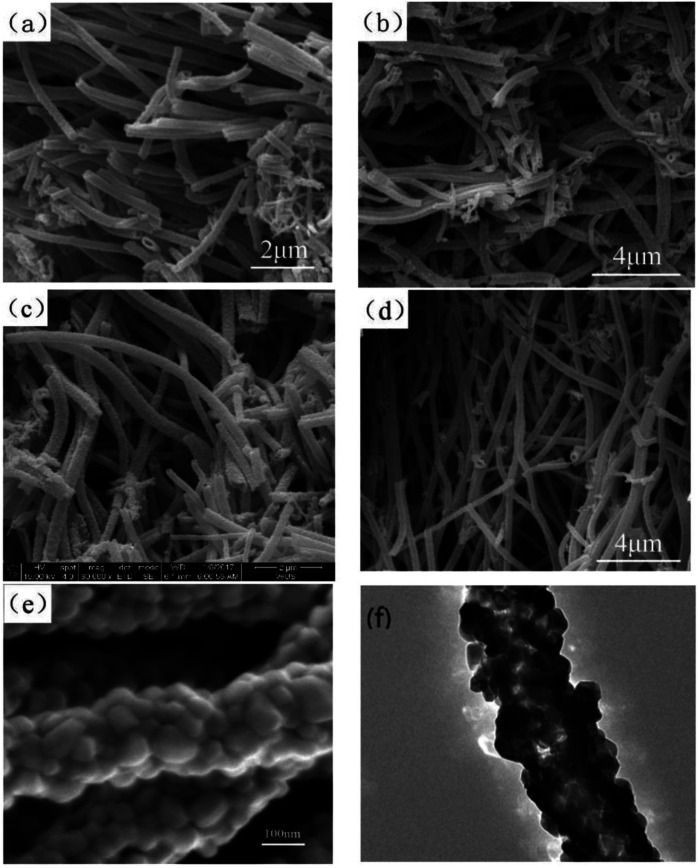
(a–c) SEM images of the pure Co_3_O_4_ nano-fibers, 20:1 sample, 20:3 sample, respectively. (d–f) SEM and TEM images of the 20:2 sample.

In addition, the authors regret that affiliation a was incorrectly shown in the original manuscript. The corrected list of affiliations is as shown above.

The Royal Society of Chemistry apologises for these errors and any consequent inconvenience to authors and readers.

## Supplementary Material

